# Supplementary material for: Tumor Recurrence and Graft Survival in Renal Transplant Recipients with a History of Pretransplant Malignancy: A Matched Pair Analysis
Source: J Clin Med. 2021 May 27;10(11):2349. doi: 10.3390/jcm10112349 (PMC8199231; doi:10.3390/jcm10112349)
Supplement: Supplementary file 1 [file jcm-10-02349-s001.zip › jcm-1206222-supplementary.pdf]

Supplementary Table S1. Matching characteristics of 1st matching group.

	PTM-RTx (*n* = 65)	RTx (*n* = 65)	*p*-Value
Age *(mean ± SD)*	62.5 ± 8.6	61.92 ± 9.1	0.099 [a]
Sex *(% males)*	36 (55.4%)	36 (55.4%)	1.000 [b]
Time under immunosuppressive drugs (days, *median, I*QR)	1825 (832, 1825)	1825 (789, 1826)	0.949 [c]

Results are presented as mean ± standard deviation (SD), median, interquartile range (IQR) ($Q_{0.25}$-$Q_{0.75}$) or relative frequencies. Categorical variables were compared using Fisher's exact test while continuous variables were compared using Student's *t*-test (normally distributed) or Mann-Whitney U test (not normally distributed). PTM = pretransplant malignancy, RTx = renal transplantation. [a] Student's *t*-test, [b] Fisher's exact test and [c] Mann-Whitney U test, *p*-value less than 0.05 was considered statistically noticeable.

Supplementary Table S2. Matching characteristics of 2nd matching group.

	PTM-RTx (*n* = 65)	RTx (*n* = 65)	*p*-Value
Age *(mean ± SD)*	62.5 ± 8.6	61.9 ± 8.6	0.156 [a]
Sex *(% males)*	36 (55.4)	38 (58.5)	0.500 [b]
Diagnosis of ESRD (*n*, %)			1.000 [b]
Hypertension	10 (15.4)	10 (15.4)	
Diabetes	6 (9.2)	6 (9.2)	
Polycystic kidney disease	9 (13.8)	9 (13.8)	
Obstructive Nephropathy	4 (6.2)	4 (6.2)	
Glomerulonephritis	19 (29.2)	19 (29.2)	
FSGS	2 (3.1)	2 (3.1)	
Interstitial nephritis	5 (7.7)	5 (7.7)	
Vasculitis	2 (3.1)	2 (3.1)	
Other	4 (6.2)	4 (6.2)	
Unknown	4 (6.2)	4 (6.2)	

Results are presented as mean ± standard deviation (SD) or relative frequencies. Categorical variables were compared using Fisher's exact test while continuous variables were compared using Student's *t*-test (normally distributed). PTM = pretransplant malignancy, RTx = renal transplantation, ESRD = end stage renal disease, FSGS = focal segmental glomerulosclerosis. [a] Student's *t*-test and [b] Fisher's exact test, a *p*-value less than 0.05 was considered statistically noticeable.

Supplementary Table S3. Baseline donor characteristics for renal transplantation recipients with and without a pretransplant malignancy, matched by age, sex and duration of immunosuppressive therapy.

	PTM-RTx (*n* = 65)	RTx (*n* = 65)	*p*-Value
Deceased donor *(n, %)*	58 (89.2)	58 (89.2)	1.000 [b]
Living donor *(n, %)*	7 (10.8)	7 (10.8)	1.000 [b]
AB0 i *(n)*	2	1	1.000 [b]
ESP *(n, %)*	25 (38.5)	24 (36.9)	1.000 [b]
Donor age *(median, IQR)*	65 (52.5, 71.5)	62 (53.0, 69.5)	0.627 [c]
Donor BMI *(median, IQR)*	26.2 (24.2, 27.9)	26 (24.4, 27.9)	0.581 [c]
Donor sex, male *(n, %)*	28 (43.1)	30 (46.2)	0.860 [b]
Donor creatinine *(median, IQR)*	1 (0.7, 1.3)	0.9 (0.6, 1.3)	0.640 [c]
KDRI (*mean ± SD)*	1.5 ± 0.6	1.4 ± 0.5	0.421 [a]
KDPI (*mean ± SD)*	76.8 ± 25.6	75.5 ± 22.5	0.758 [a]

CIT (h) *(mean ± SD)*	9.9 ± 4.5	9.5 ± 4.9	0.612 [a]
WIT (min) *(mean ± SD)*	31.8 ± 7.4	32.9 ± 6.9	0.373 [a]
# HLA mismatch *(mean ± SD)*	3.3 ± 1.6	3.1 ± 1.8	0.550 [a]
# HLA-A mismatch *(% 0/1/2)*	30.8/53.8/15.4	30.8/40.0/29.2	0.261 [b]
# HLA-B mismatch *(% 0/1/2)*	13.8/41.5/44.6	18.5/47.7/33.8	0.231 [b]
# HLA-DR mismatch *(% 0/1/2)*	23.1/41.5/35.4	30.8/46.2/23.1	0.145 [b]

Results are presented as mean ± standard deviation (SD), median, interquartile range (IQR) ($Q_{0.25}$–$Q_{0.75}$) or relative frequencies. Categorical variables were compared using Fisher's exact test while continuous variables were compared using Student's *t*-test (normally distributed) or Mann-Whitney U test (not normally distributed). PTM = pretransplant malignancy, RTx = renal transplantation, ESP = European Senior Program, AB0i = AB0-incompatible transplantation, BMI = body mass index, KDRI = Kideny Donor Risk Index, KDPI = Kidney Donor Profile Index, CIT = cold ischemia time, WIT = warm ischemia time, HLA = Human Leukocyte Antigen. [a] Student's *t*-test, [b] Fisher's exact test and [c] Mann-Whitney U test, a *p*-value less than 0.05 was considered statistically noticeable.

Supplementary Table S4. Baseline recipient characteristics for renal transplantation recipients with and without a pretransplant malignancy, matched by age, sex, and duration of immunosuppressive therapy.

	**PTM-RTx (*n* = 65)**	**RTx (*n* = 65)**	***p*-Value**
Age *(mean ± SD)*	62.5 ± 8.6	61.9 ± 9.1	1.000 [a]
Sex *(% males)*	55.4	55.4	1.000 [b]
BMI *(kg/m^2, mean ± SD)*	25.4 ± 3.3	25.6 ± 4.5	0.725 [a]
Cause of ESRD (*n*, %)			0.738 [b]
Hypertension	10 (15.4%)	10 (15.4%)	
Diabetes	6 (9.2%)	4 (6.2%)	
Polycystic kidney disease	9 (13.8%)	12 (18.5%)	
Obstructive nephropathy	4 (6.2%)	4 (6.2%)	
Glomerulonephritis	19 (29.2%)	19 (29.2%)	
FSGS	2 (3.1%)	3 (4.6%)	
Interstitial nephritis	5 (7.7%)	4 (6.2%)	
Vasculitis	2 (3.1%)	1 (1.5%)	
Other	4 (6.2%)	7 (10.8%)	
Unknown	4 (6.2%)	1 (1.5%)	
RRT *(n, % yes)*	64 (98.5)	62 (95.4)	0.625 [b]
Dialysis vintage *(d, mean ± SD)*	1841.9 ± 962.4	2014.6 ± 1071.6	0.124 [a]
Hypertension *(n, %)*	59 (90.8)	63 (96.9)	0.289 [b]
Diabetes *(n, %)*	11 (16.9)	9 (13.8)	0.804 [b]
CAD *(n, %)*	18 (27.7)	19 (29.2)	1.000 [b]

Results are presented as mean ± standard deviation (SD) or relative frequencies. Categorical variables were compared using Fisher's exact test while continuous variables were compared using Student's *t*-test (normally distributed). PTM = pretransplant malignancy, RTx = renal transplantation, BMI = body mass index, ESRD = end stage renal disease, FSGS = Focal Segmental Glomerulosclerosis, RRT = renal replacement therapy, CAD = coronary artery disease. [a] Student's *t*-test and [b] Fisher's exact test, a *p*-value less than 0.05 was considered statistically noticeable.

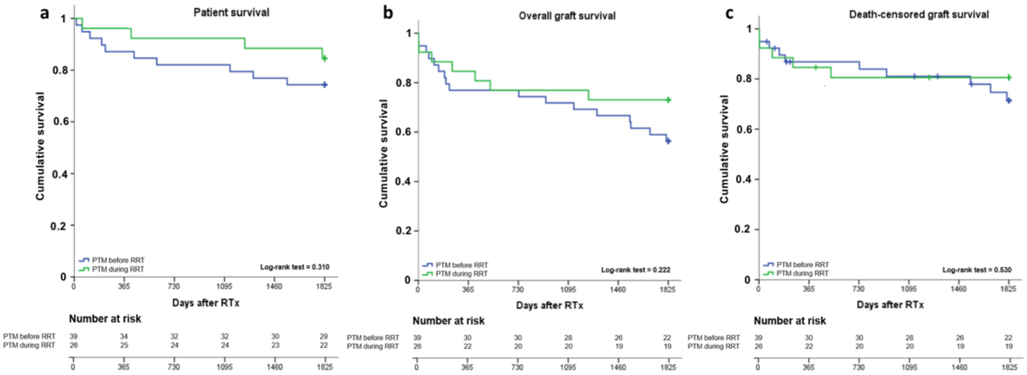

Supplementary Figure S1. Kaplan-Meier curves for five-year patient and graft survival in pretransplant malignancy (PTM) renal transplantation (RTx)-recipients stratified for PTM diagnosis before and during renal replacement therapy (RRT). Longitudinal patient (**a**), overall graft (**b**), and death-censored graft survival (**c**) stratified for RTx-recipients who developed PTM before or during RRT. Survival rates of RTx-recipients with PTM during RRT (green lines) or PTM before RRT (blue lines) were estimated by Kaplan-Meier methodology and compared by log-rank test.

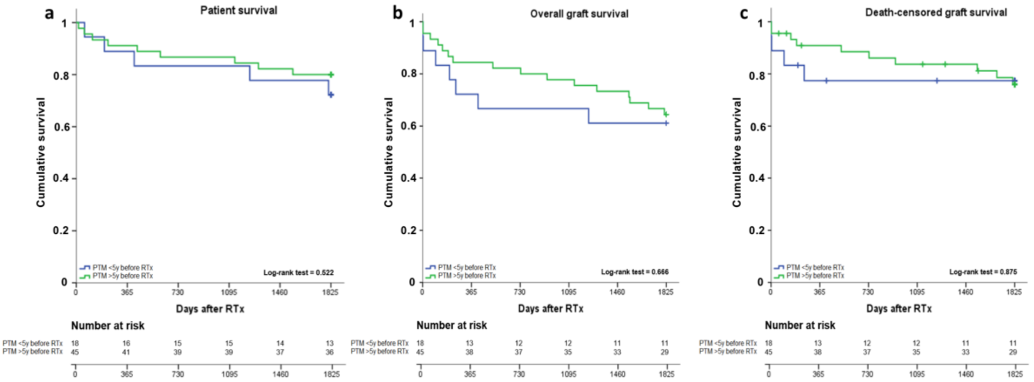

Supplementary Figure S2. Kaplan-Meier curves for patient and graft survival in pretransplant malignancy (PTM) renal transplantation (RTx)-recipients stratified for PTM diagnosis more or less than five years before renal transplantation. Longitudinal patient (**a**), overall graft (**b**) and death-censored graft survival (**c**) stratified for RTx-recipients who developed PTM more than five years before and less than five years before RTx. Survival rates of RTx-recipients with PTM > 5 years before RTx (green lines) and RTx-recipients with PTM < 5 years before RTx (blue lines) were estimated by Kaplan-Meier methodology and compared by log-rank test.
